# Decision rules for determining terrestrial movement and the consequences for filtering high-resolution global positioning system tracks: a case study using the African lion (*Panthera leo*)

**DOI:** 10.1098/rsif.2021.0692

**Published:** 2022-01-19

**Authors:** Richard M. Gunner, Rory P. Wilson, Mark D. Holton, Phil Hopkins, Stephen H. Bell, Nikki J. Marks, Nigel C. Bennett, Sam Ferreira, Danny Govender, Pauli Viljoen, Angela Bruns, O. Louis van Schalkwyk, Mads F. Bertelsen, Carlos M. Duarte, Martin C. van Rooyen, Craig J. Tambling, Aoife Göppert, Delmar Diesel, D. Michael Scantlebury

**Affiliations:** ^1^ Department for the Ecology of Animal Societies Radolfzell, Max Planck Institute of Animal Behavior, Baden-Württemberg, Germany; ^2^ Department for the Ecology of Animal Societies, Max Planck Institute of Animal Behavior, 78315 Radolfzell, Germany; ^3^ School of Biological Sciences, Queen's University Belfast, 19 Chlorine Gardens, Belfast BT9 5DL, UK; ^4^ Mammal Research Institute, Department of Zoology and Entomology, University of Pretoria, Pretoria 002, South Africa; ^5^ Savanna and Grassland Research Unit, South African National Parks, Scientific Services Skukuza, Kruger National Park, Skukuza 1350, South Africa; ^6^ Veterinary Wildlife Services, South African National Parks, 97 Memorial Road, Old Testing Grounds, 8301 Kimberley, South Africa; ^7^ Department of Agriculture, Forestry and Fisheries, Government of South Africa, Skukuza, South Africa; ^8^ Department of Migration, Max Planck Institute of Animal Behavior, 78315 Radolfzell, Germany; ^9^ Center for Zoo and Wild Animal Health, Copenhagen Zoo, Roskildevej 38, 2000 Frederiksberg, Denmark; ^10^ Red Sea Research Centre, King Abdullah University of Science and Technology, Thuwal 23955, Saudi Arabia; ^11^ Department of Zoology and Entomology, University of Fort Hare Alice Campus, Ring Road, Alice 5700, South Africa

**Keywords:** acceleration, animal behaviour, data filtering, global positioning system, highresolution, terrestrial movement

## Abstract

The combined use of global positioning system (GPS) technology and motion sensors within the discipline of movement ecology has increased over recent years. This is particularly the case for instrumented wildlife, with many studies now opting to record parameters at high (infra-second) sampling frequencies. However, the detail with which GPS loggers can elucidate fine-scale movement depends on the precision and accuracy of fixes, with accuracy being affected by signal reception. We hypothesized that animal behaviour was the main factor affecting fix inaccuracy, with inherent GPS positional noise (jitter) being most apparent during GPS fixes for non-moving locations, thereby producing disproportionate error during rest periods. A movement-verified filtering (MVF) protocol was constructed to compare GPS-derived speed data with dynamic body acceleration, to provide a computationally quick method for identifying genuine travelling movement. This method was tested on 11 free-ranging lions (*Panthera leo*) fitted with collar-mounted GPS units and tri-axial motion sensors recording at 1 and 40 Hz, respectively. The findings support the hypothesis and show that distance moved estimates were, on average, overestimated by greater than 80% prior to GPS screening. We present the conceptual and mathematical protocols for screening fix inaccuracy within high-resolution GPS datasets and demonstrate the importance that MVF has for avoiding inaccurate and biased estimates of movement.

## 1. Introduction

A popular method to determine terrestrial animal movement uses global positioning system (GPS) technology, which enables long-term continuous spatial monitoring of wild animals without disturbing them (for reviews see [1,2–5]). This approach has led to broad applications, including examination of home ranges [6,7], migratory routes [8–10], habitat use [11,12], resource allocation [13,14], activity budgets [15–17] as well as social interactions [18]. Since their inception, animal-borne GPSs have reduced considerably in mass and size, while data storage capacity, battery longevity and affordability have improved [5,19,20]. Consequently, scientists can now track animals as small as *ca* 20 g songbirds (*Seiurus aurocapilla*) [21] at frequencies as high as 10 Hz (e.g. [22]), providing so much detail of animal movement that even animal behaviour can often be inferred [23–25]. Such inference is, however, limited by fix precision, regardless of fix accuracy, which can be particularly ambiguous when the movement rates of the focal species are less than the spatial resolution of the GPS fixes [26]. Species-specific resampling strategies and correction factors can go some way to redressing this (see [26,27–29]).

Many factors affect GPS performance, including habitat type and heterogeneity [30–33], topography of the terrain [34,35], clear sky availability [36], weather conditions [31], submersion in water [37,38], time of day [39], vegetation cover/type [34,40], GPS orientation [41] and fix acquisition rate [42,43], in addition to the number of available satellites and their orbiting geometry with respect to one another [44,45]. All these elements affect the propagation of signal quality and/or receiver reception capability and thus increase triangulation error (see Hofman *et al.* [4] for review), often assessed via the dilution of precision (DOP) values [45,46].

Species-specific movements can be misinterpreted because GPS error often exaggerates the extent of movement, with error associated with distance measures being additive over time, and particularly germane at higher sampling frequencies (given that higher rates of error are incorporated per unit time) [43,47,48]. Indeed, although a number of authors have attempted to resolve the accuracy of GPS performance by quantifying the fix success rate and location error over various scenarios (see [49,50]), the critical modulator of GPS performance is animal behaviour (see [41,51–54]). For example, Heard *et al.* [39] demonstrated that fix success rate for GPS collars on grizzly bears (*Ursus arctos*) followed a bimodal circadian pattern, which was paralleled to the activity time budgets of the bear, with higher forest density cover and variability in collar orientation being attributed to declines in fix rate. Similarly, after collaring both Eurasian lynx (*Lynx lynx*) and wolverine (*Gulo gulo*) in a similar habitat, Mattisson *et al.* [52] suggested that high discrepancy in fix rate between the two species could be explained by differences in their behavioural repertoire. In essence, the specifics of animal movement, the ‘what', ‘where’, ‘when' and ‘how' (see [1]), underpins the species interaction with its environment and consequently the dual proficiency of signal propagation and reception between satellites and receiver. Resting is the most common behaviour for most terrestrial animals (particularly carnivores) and critically affects the fix accuracy, because resting is typically associated with a change of body position (e.g. resting on the collar) and/or coverage within/near ‘signal obstructing' environmental features (e.g. sleeping under trees or in caves/burrows), thus decreasing the available sky for the GPS receiver [32–34,45]. This issue is compounded for collar-mounted GPS devices, because behaviours variously affect the position of the GPS antenna even though many collars are designed to be bottom-weighted to minimize this problem [34,51,55].

Despite the well-documented issues of locational error and numerous mitigation strategies being proposed [56–59], there has been no ‘gold standard' solution to identify inaccurate fixes. For example, Lewis *et al.* [44] emphasized using DOP values and removing fixes with values greater than 5 and only keeping positions where three or more satellites were registered to eliminate potentially large location errors. This recommendation was based on the premise that a wider geometry of satellite spacing results in lower recorded DOP values and this, along with a higher number of registered satellites, is associated with minimizing triangulation errors. The relationship between spatial precision and increasing DOP values, while generally accepted, is noisy and can reduce datasets considerably, while still leaving notably anomalous fixes intact [35,60]. Juxtaposed to this, Bjørneraas *et al.* [49] developed a method that focused on the movement characteristics of the focal species to identify large locational errors with minimal data reduction. This included screening for unrealistic distances travelled, speeds and turn angles between successive locations. However, this can become complicated and arbitrary at high sampling frequencies and is computationally intensive for large datasets.

To our knowledge, a specific solution for screening inaccurate locations from high-resolution GPS data (e.g. ≥1 Hz) has not yet been proposed. The difficulty is that, while shorter fix intervals are typically associated with higher fix accuracy [42,51,61], locational error is, within the wider context of daily movement, relatively small and harder to identify accurately. Disentangling this error is particularly relevant because GPS units used on animals with high fix rates are usually deployed with fine-scale analysis of movement trends in mind (see [26]).

We note that since GPS ‘jitter' (a term we use to define fixes inaccurately fluctuating around a central location) is disproportionately high during stationary periods [38,42,47] the viability of deriving accurate movement from high-resolution GPS trajectories depends on the ability to determine when an animal is moving or not in a manner that is independent of the GPS-derived movement. Studies have already used acceleration to activate GPS units only during movement, both as a means to increase battery longevity and to avoid the fix inaccuracy prevalent during periods of inactivity [53,62]. Properly coupled GPS–acceleration systems are uncommon however, because a moving animal (as discerned from the accelerometer) does not necessarily correspond with a working GPS (e.g. due to signal obstruction and because cold start ‘blind' satellite searches are associated with lower fix success rates [42]). For highly resolved animal tracks, we advocate the importance of recording fixes continuously, in part to mitigate performance issues associated with cold starts between fix intervals [26,42,63] but also because fine-scale GPS estimates can be compared alongside acceleration data to aid in differentiating between non-travelling movements and travelling movements (see [64]). Beyond this, identifying ‘hotspots' of GPS jitter may be useful for discerning GPS performance according to habitat type and/or behaviour. As part of this, we propose a new method for screening raw, high-resolution GPS data by accounting for the amount of activity using accelerometers and equating their outputs with an estimate of speed to evaluate the likelihood of movement per unit time. This is based on the observation that dynamic body acceleration (DBA—for definition see Wilson *et al.* [65]) increases approximately linearly with speed in terrestrial animals [66–68]. Thus, any GPS-derived speed should co-vary with DBA.

Here, we propose a decision tree-based framework in which user-defined thresholds of (i) GPS speed, (ii) DBA, and (iii) time are implemented to screen GPS fixes and remove those that do not equate to genuine travelling movement. We also suggest an initial method for screening extreme anomalous fixes using distance estimates between the raw GPS track and the median filtered equivalent. We illustrate this using data from 11 GPS collar-fitted free-ranging lions (*Panthera leo)* within the Kgalagadi Transfrontier Park in the Kalahari Desert. The aims of this study are to provide both the conceptual and methodological protocol for screening high-resolution GPS data using a movement-verified filtering (MVF) protocol and to discuss the broader applicability this method has for discerning animal movement.

## 2. Methods

The procedure was applied to 14 days of data derived from 11 wild lions (five males and six females) in the Kgalagadi Transfrontier Park, South Africa, during February–March 2019. Lions were equipped with a LiteTrack GPS collar (Lotek Wireless Inc. [69]), to which a Gypsy_5 Techno-smart GPS unit (Technosmart s.r.l. [70]) set to record at 1 Hz and a ‘Daily Diary’ (DD) (containing *inter alia* tri-axial accelerometers and tri-axial magnetometers) (see [71]) recording at 40 Hz were attached. The GPS units were encased in a thick 3D-printed acrylonitrile–butadiene–styrene (ABS) plastic oval housing and DDs were enclosed in a water-tight aluminium housing (see electronic supplementary material, figure S1.1). In total, 15 lions from four prides between 19 and 25 February 2019 were collared. Twelve collars were fitted with the Gypsy_5 Techno-smart GPS units and all collars were fitted with DDs. However, one DD (which was paired with a Gypsy_5 unit) malfunctioned, so 11 complete DD-Gypsy_5 datasets were analysed in this study. There were two collar sizes: small collars weighed 1.24 kg and large collars weighed 1.33 kg (attached with all devices), which constituted less than 2% and less than 1% of the body mass of the lightest equipped female and male animals, respectively. Lions were recaptured two weeks after the initial deployment to retrieve the Gypsy_5 GPS and replace the DD SD cards. The collars remained on the lions as part of a longer term study, releasing automatically using an on-board timed drop-off mechanism—later found using the VHF beacon. See electronic supplementary material, S1 for more information on the study site, capture protocol and devices used. All analyses were performed in Daily Diary Multi Trace (DDMT) [72], R (v. 3.6.2, [73]) and Origin pro 2016 (OriginLab Corporation, [74]).

Intermittent behavioural observations of each pride took place at dawn and dusk, and occasionally during the day and night, for approximately 2–3 h. During these periods, ethograms of the collared individual's various activities were recorded to document movement for comparison with the acceleration and GPS speed estimates to verify the accuracy of our MVF thresholds (table 1). These observations were also performed to check for any potential negative side effects of the collars, though none were apparent.

**Table 1.** Contingency table documenting the mean accuracy and misclassification rate of the MVF method from∼25 h of behavioural observations (ethograms) between eight individuals. FN, false negative; FP, false positive; TN, true negative; TP, true positive.

		test data (actual)		accuracy (TP + TN/TP + TN + FP + FN)
		positive (moving)	negative (non-moving)	
predicted (MVF method)	positive (MVF = 1 = moving)	true positive rate (TPR) $\;\mathrm{TPR}=\frac{\mathrm{TP}}{\left(\mathrm{TP}\;+\;\mathrm{FN}\right)}\times 100=95.21\text{\%}$	false positive rate (FPR) $\mathrm{FPR}=\frac{\mathrm{FP}}{\left(\mathrm{FP}+\mathrm{TN}\right)}\times 100=0.35\text{\%}$	97.43%
	negative (MVF = 0 = non-moving)	false negative rate (FNR) $\mathrm{FNR}=\frac{\mathrm{FN}}{\left(\mathrm{FN}+\mathrm{TP}\right)}\times 100=4.79\text{\%}$	true negative rate (TNR) $\mathrm{TNR}=\frac{\mathrm{TN}}{\left(\mathrm{TN}+\mathrm{FP}\right)}\times 100=99.65\text{\%}$	
test data (actual)	time spent moving/ non-moving	19.37%	80.63%	
	VeDBA (±1 s.d.)	0.198 ± 0.058	0.039 ± 0.012	

### 2.1. The movement-verified filtering method

**Figure 1.**
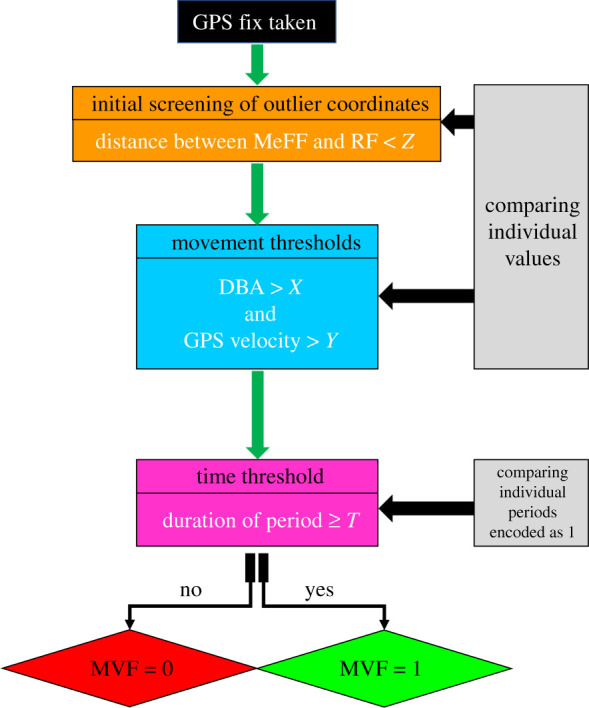
Schematic of the derivation of MVF. GPS fixes with an MVF value of 1 are considered to be more accurate given that the data indicate travelling. Note that values used at each stage (including the stepping range and post-smoothing windows in the prior derivations of GPS speed and VeDBA) are user defined and must be adapted for the study species.

The MVF protocol (illustrated in figure 1) primarily involves deriving DBA from tri-axial accelerometery data, computing speed from GPS data and evaluating how both covary during travelling movement. The user then decides on the threshold limits that DBA and GPS speed must exceed (in terms of both magnitude and duration) for a movement bout to be verified. Specifically, the step-by-step method (used for lions) is set out in §§2.1.1–2.1.6.

#### 2.1.1. Derivation of DBA

Vectorial dynamic body acceleration (VeDBA) [75] was the DBA metric used for activity [65] and as a proxy for speed [66]. VeDBA is the vectorial sum of the DBA in a tri-axial acceleration signal (see electronic supplementary material, S2). A rolling mean was applied to raw VeDBA values (a 2 s centre-aligned window was used for lions) to ensure that both acceleration and deceleration components of an animal's stride cycle were incorporated together within any particular time period [65].

#### 2.1.2. Derivation of GPS speed

The trigonometric Haversine formula [76,77] was used to calculate the shortest distance between fixes of an appropriate stepping range (see electronic supplementary material, S2). We define a stepping range as the interval between each retained fix: a five-fix stepping range was used for lions (distance computed between every fifth fix). Each successive distance estimate was divided by its time period (between retained fixes) to convert to GPS speed (m s^−1^). A rolling mean was applied to GPS speed (5 s centre-aligned rolling mean used for lions) for greater interpolation purposes with respect to acceleration estimates (see Discussion and electronic supplementary material, S2, detailing the importance of a suitable stepping/post-smoothing range). Missing fixes were not included in the computation of GPS speed.

#### 2.1.3. Time synchronizing GPS speed and DBA data

Both VeDBA and GPS speed data were time synchronized and sub-sampled to 1 Hz to make the data more manageable for analysis and because differentiating between fine-scale behaviours was not a prime objective of this study. Missing locational data were expressed as ‘NA’.

#### 2.1.4. Using GPS-derived distance to identify extreme outliers: distance threshold (*Z*)

Missing locational data were replaced by linear interpolation between fixes (we define this set of coordinates as ‘raw fixes’; RF). To identify extreme outliers, a median rolling filter was applied to both the longitude and latitude coordinates of the RF (we define this set of coordinates as ‘median filtered fixes’; MeFF). The Haversine method was then used to calculate the distance (units in metres) between the two sets of coordinates (RF versus MeFF) per unit time. Locational data (RF) above the *Z* threshold were deemed outliers (and thus failed the first step of the MVF protocol). By applying a rolling median using a suitable window length, large distance estimates reflecting either single or multiple ‘batched' outlier(s) could be distinguished from fixes deemed ‘accurate' but highly separated in space owing to large gaps in locational data. The window length size and *Z* threshold should be chosen according to the animal in question because of the scales of movement undertaken by different species (median filter window length of 60 s and a lenient threshold of 100 m used for lions). The window length should be large enough so that the calculated median is not affected by a potential batch of consecutive anomalies at any one time. When plotted against time, the distance between RF and MeFF shows relatively consistent variation about a given range (dependent on the window size set), though large obvious spikes indicate outliers, and the extent of this disparity can give an indication of the *Z* threshold to set.

#### 2.1.5. Movement thresholds (*X* and *Y*)

The second stage for screening the GPS data was the thresholds of VeDBA (*X*_VeDBA_) and GPS speed (*Y*_GPS_) that infer moving behaviour. We set the protocol for fixes to fail the MVF protocol when:

(i) VeDBA < *X* and GPS speed > *Y* (likely resultant from locational error)
(ii) VeDBA > *X* and GPS speed < *Y* (likely resultant from a stationary behaviour),

where *X* and *Y* were given defined thresholds.

For the lions, after initial inspection of data with respect to ground-truthed behavioural observations, the threshold *X* was determined as 0.11 *g* and the threshold *Y* was determined as 0.35 m s^−1^ (see below). These thresholds were lenient, incorporating even slow movement and accounting for discrepancies of the relative magnitude of acceleration estimates between individuals (see [65,78]).

#### 2.1.6. Time threshold (*T*)

The final stage of validating movement was to implement a minimum time threshold (*T*), over which uninterrupted movement had to occur before it was classified as such. This was implemented to discern travelling movement (where the animal location changed) from non-travelling movement (e.g. when the animal rolled over) for periods when both *X*_VeDBA_ and *Y*_GPS_ thresholds were met. MVF values were assigned a value of 1, for every GPS fix that was time-matched to periods where the above thresholds (*X*_VeDBA_ and *Y*_GPS_) were met for a minimum duration of *T* (5 s was used for lions in the current study). MVF periods encoded as 1 occurring ≤ 2 s from one another were merged. An MVF value of 0 represented either missing locational data, extreme outliers (identified by Z threshold) or periods when the data indicated the animal was non-moving.

### 2.2. Data analyses

Various movement-derived metrics were compared between periods when animals were deemed to be moving (‘travelling' movement; MVF = 1) and periods when they were deemed to be non-moving (‘non-travelling'/stationary movement; MVF = 0). Such metrics include estimates of pitch, roll, heading, distance travelled, speed and tortuosity estimates (see electronic supplementary material, S4 for procedures and references therein). Here, unless otherwise stated, data ascribed as non-moving do not include data when GPS positions were missing or were extreme outliers (the latter determined by the *Z* threshold as described above). Results presented as percentages are given as ‘x' with variance as one standard deviation (s.d.) and range in the format ($\bar{x}$ ± 1 s.d. (range_min_ − range_max_)).

## 3. Results

Across 25 h of behavioural observations, the MVF method using the thresholds outlined above registered an average accuracy of 97% (table 1; data correctly assigned as moving). This protocol was determined to have a high true negative rate (greater than 99%) and low false positive rate (less than 1%), indicating that data that surpassed the MVF protocol indeed showed that the animal was moving with a high degree of certainty. The true positive rate was slightly lower (*ca* 95%) and was perceived to have been primarily modulated according to the variability in fix latency, which (irrespective of stepping/post-smoothing range) can result in a time delay, uncoupling estimates of GPS speed from the instantaneous and definitive expression of DBA estimates. It thus occasionally results in the beginning or end of periods that animals were moving being misclassified as ‘non-moving' (MVF = 0).

**Figure 2.**
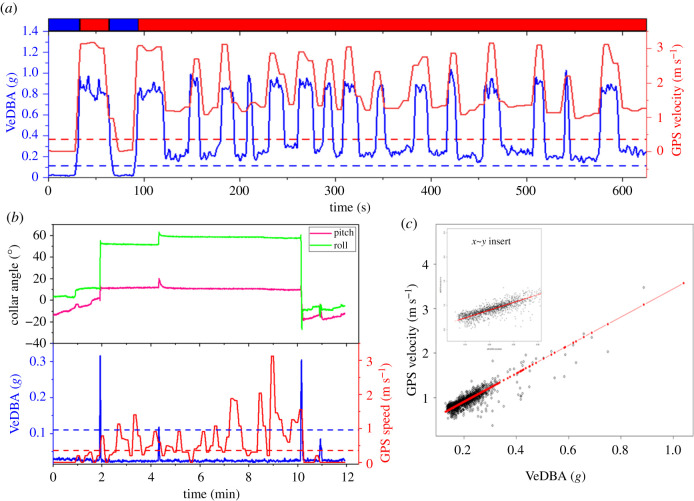
Example of the movement-based thresholds. (*a*) A period of predominantly continuous movement (coloured rug at the top of plot denotes MVF values (1 = moving (red), 0 = non-moving (blue)). The peaks of both VeDBA and GPS velocity are due to bouts of running, interspaced by either non-moving or walking bouts. (*b*) Relationship between VeDBA and GPS speed during a rest period, whereby the individual carried out a transitionary roll while lying prone (at approx. the 2 min mark; as depicted by the pitch and roll angles), after which GPS jitter became more apparent (as demonstrated by the higher variance in GPS speed estimates). (*c*) GPS speed∼VeDBA relationship for a given lion with linear regression (*y* = *a* + *bx* and zoomed in the inset). Data from (*c*) are taken only from marked moving periods following the MVF method. Each data point represents the mean value per period, taken from *ca* two weeks of data acquisition.

**Figure 3.**
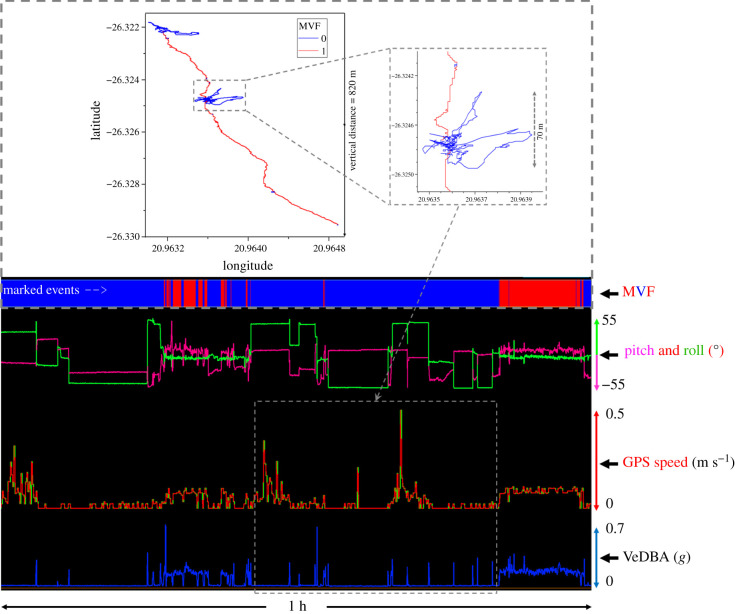
DD- and GPS-derived data showing intermittent periods of moving and stationary behaviours (lower panel = two-dimensional waveforms versus time of: VeDBA, GPS speed (raw = red, green = smoothed) and pitch and roll collar angles; upper panel = GPS fixes coloured according to MVF values (MVF = 0 = blue; ‘non-moving' | MVF = 1 = red; ‘moving')). Note how many of the periods determined as non-moving (MVF = 0) had high estimates of GPS speed owing to large locational errors and this often followed sharp peaks in VeDBA, coinciding with a postural change (non-travelling behaviour). Note also how closely GPS speed estimates follow the VeDBA trace during periods of predominantly moving (MVF = 1) and the consistency of pitch and roll values (with intermittent bouts of stationary behaviour associated with a change in collar angle). The magnified insert in the upper panel exemplifies the high vertical and horizontal straight-line distance between track coordinates due to GPS jitter.

**Figure 4.**
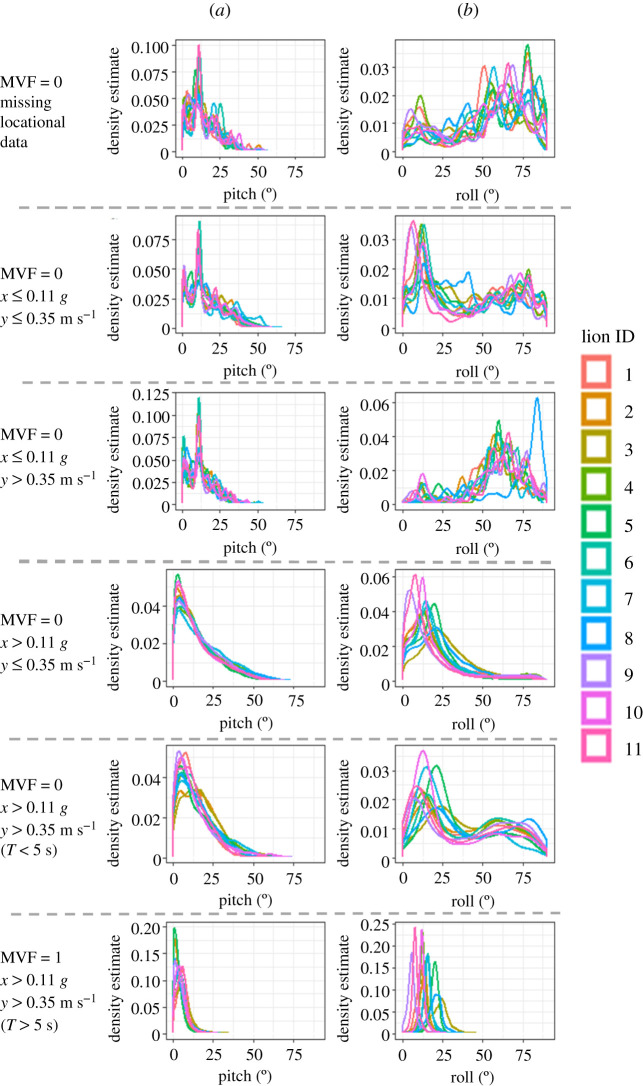
Indices of collar postural offsets per lion, assessed via density estimates of absolute values of (*a*) pitch and (*b*) roll. Plots are facetted row-wise according to five scenarios as described to the left of each plot row. The distributions become smoother and unimodal at higher levels of activity.

Fix success rate for the GPS varied between 89% and 97% across different animals. There was no indication of systematic drop-out (variability of fix success rate) being modulated according to time over the 14 day monitoring periods (electronic supplementary material, table S3.1 and figure S3.1). Generally, GPS-derived speed correlated well with VeDBA ($\bar{x}$ *r^2^* = 0.74 ± 0.04 (0.67–0.81)) (electronic supplementary material, figure S2.4), especially during periods that were defined by the MVF protocol as ‘movement' (figure 2*a,c*; electronic supplementary material, figure S2.1:3). Discrepancies between GPS speed and VeDBA were associated with location error (figure 3; electronic supplementary material, figure S2.3), with the MVF approach highlighting that the position of the collar depended on the animal's behaviour (figure 4; electronic supplementary material, table S3.2) and that this was a prime modulator of GPS performance (cf. figure 3 and figure 2*b*; electronic supplementary material, figure S2.3).

**Figure 5.**
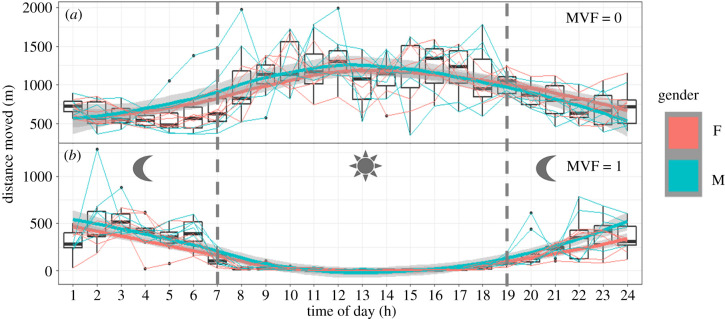
Mean summed distance moved (m) per hour per individual (see electronic supplementary material, S4 for a full description of methods). Each individual's hourly mean is connected across time via a straight line (coloured according to gender; red = female, blue = male). Plots are fitted with a line of best fit according to gender, using a ‘gam smoothing' (grey shading around the line represents the 95% confidence level interval). This procedure was applied independently for non-moving (*a*) and moving (*b*) individuals. Note the disparity in distance estimates, with non-moving bouts demonstrating high values during sunlight hours (approx. between 7.00 and 19.00 (grey bars)).

On average, 13.3% ± 3.3 (8.3–19.5) of data acquisition passed the MVF protocol (electronic supplementary material, table S3.2). The majority of data deemed to be non-moving, 70.4% ± 3.6 (65–77), was due to both *X*_VeDBA_ and *Y*_GPS_ thresholds not being met. However, an appreciable proportion of non-moving data was due to the *Y*_GPS_ threshold being met, but not the *X*_VeDBA_ threshold, 12.4% ± 3.0 (9–18), or both *Y*_GPS_ and *X*_VeDBA_ thresholds being met, but not for the duration of *T*_time_, 12.5% ± 2.9 (8–18). Data where *X*_VeDBA_ was met, but not *Y*_GPS_, comprised 4.85% ± 1.3 (3–7) (electronic supplementary material, figure S3.2). The additive nature of errors associated with GPS jitter was significant and exemplified within cumulative distances moved (between fixes) (figure 5; electronic supplementary material, table S3.2) and was apparent even at the broadest scales of movement (electronic supplementary material, figure S2.5). It was clear that GPS jitter was much more prominent when lions were resting; unless these data were filtered, use of these raw unfiltered GPS data resulted in biased and erroneous speed, distance and tortuosity of movement estimates (electronic supplementary material, table S3.2). Following the MVF method, there appeared to be a greater correlation between DD- and GPS-derived heading estimates (electronic supplementary material, figure S4.1).

## 4. Discussion

### 4.1. Evaluation of the MVF protocol

This work demonstrates the value of using both DBA and GPS data to discern moving behaviours from stationary behaviours with a computationally quick protocol which effectively filters inaccurate fixes from high-frequency GPS data (e.g. ≥1 Hz, though it should work at lower frequencies; see electronic supplementary material, figures S2.1 and S2.2). The central premise is that when the magnitude of GPS speed and VeDBA both indicate movement (via pre-set thresholds), then movement is indeed likely (table 1, figure 3 and figure 2*a*; electronic supplementary material, figures S2.1 and S2.3). This highlights the problem of GPS jitter when VeDBA does not correspond to movement even though the GPS indicates otherwise. Conversely, (relatively energetic) non-travelling behaviours are flagged when the magnitude of VeDBA infers movement while data on GPS speed do not.

Our results reaffirm the importance of screening GPS inaccuracies within high-frequency independently collected datasets of animal movements, owing to the additive nature of GPS jitter, which is most prevalent during rest periods (figure 3; electronic supplementary material, figure S2.3 and table S3.2). This was particularly relevant in the current study because of the high proportion of data allocated to non-moving behaviours (electronic supplementary material, table S3.2) (reflecting the energy-conservation strategy that Kalahari lions adopt (see [79]). Indices of collar/postural offsets (evaluated using absolute values of pitch and roll) showed high variability during times when GPS units did not acquire fixes (figure 4), even when the fix success rate could not be attributed to battery longevity (electronic supplementary material, table S3.1, figure S3.1). Animal behaviour (including habitat selection) thus seems to be a primary factor affecting fix success rate and quality. Clear mono-modal peaks in the indices of posture were only witnessed when all thresholds of our MVF approach were met (figure 4). While there were slight differences in the tightness of these distributions between lions (presumably because of discrepancies between collar fit), this does suggest that the optimum collar–body position for acquiring satellite signals occurred during travelling movement. By contrast, distributions were much more varied during times of non-moving, again highlighting the interplay between animal behaviour, collar orientation and GPS performance.

Our results highlight how, in the absence of appropriate filtering, inappropriate conclusions about a species’ movement can be made. Here, there were stark contrasts of tortuosity, speed and, most notably, distance travelled estimates between sets of data that both passed and failed our MVF method (electronic supplementary material, table S3.2). This method may therefore have particular value for distinguishing true small-scale area-restricted search (ARS) behaviour [80] by removing spurious turn angles caused by jitter [81,82] (see electronic supplementary material, figure S4.1). Here, cumulative distance from non-moving data was 80% higher than their actual moving periods for some lions. This highly inflated index of movement was exemplified when measured as hourly averages (figure 5), apparently showing that lions travelled greater distances during the hottest parts of the day, something that is extremely unlikely (see [83]). Furthermore, our MVF protocol reduced the apparent maximum speed of any lion from greater than 150 to 48 km h^−1^. This critical issue highlights the drawbacks of assessing GPS data sampled at high frequency (in spite of necessary post-resampling strategies (electronic supplementary material, S2)), which intensifies erroneous location estimates (see figure 3 and electronic supplementary material, figure S2.3), even at macro-scales of movement (electronic supplementary material, figure S2.5).

### 4.2. Utility of the MVF protocol according to species-specific and environmental circumstance

The Haversine method for determination of animal speed and location using GPS positional fixes can estimate distances travelled with high precision [76]. However, for datasets containing many points collected at high frequencies, distance estimates are unreliable at small stepping ranges owing to the interplay between location error and the precision of longitude and latitude coordinates that produce additive errors [26,84]. Most commercial GPS units record fixes to five decimal places, with the fifth digit of the decimal place giving approximately 1.1 m resolution. Furthermore, the computation time for a device to record a GPS fix can vary, reducing the synchronization of time between both GPS and the accelerometer logger. Given that many terrestrial animals maintain relatively low travel speeds for extended periods (see [85]), we note that an appropriate choice of stepping range and smoothing window is critical for deducing reasonable step-length estimates per unit time (electronic supplementary material, figures S2.1 and S2.2), with this being dependent upon the (species-specific) scales of movement being assessed (see [29,66,86]).

Essentially, there is a trade-off between incorporating higher rates of (precision-based) error at smaller stepping ranges and increasing the lag of change relative to the properly time-synchronized acceleration data at higher stepping ranges. This means that accurate fine-scale estimates of GPS-derived speed are not possible and so the relationship with body movement measurements such as VeDBA will never be succinct given the disparity of resolution from both measures. In addition, inter- and intra-specific variations of acceleration estimates can arise owing to discrepancies of: morphology [66], locomotion mechanisms (e.g. change in gait to facilitate higher speeds [87]), extrinsic factors (e.g. moving over a deformable substrate/changeable grade [86,88]), tag placement [65] and collar roll [89,90], thereby altering the relationship between VeDBA and mechanical power (and thus speed) [86,91].

Alongside GPS resampling, MVF user-defined thresholds are expected to change according to the study species and scales of movement in question. For example, DBA estimates (specifically ‘overall dynamic body acceleration'; ODBA [65]) of African elephants (*Loxodonta africana*) typically ranged between 0.15 and 0.3 *g* during periods of walking [92] and this is comparable to that reported for Eurasian beavers (*Castor fibre*) (0.265 ± 0.029) [93]. Though, notably, both species have different leg lengths and move with very different gaits, which gives very different DBA-dependent speed estimates, as demonstrated by Bidder *et al.* [66] for multiple species.

It is notable here that we have focused on terrestrial movement, and this is primarily because the relationship between DBA and speed can break down substantially for many aquatic and aerial species. This occurs because, for example, birds can glide at a variety of ground speeds (depending on, for example, wind vectors and glide angle) without changing DBA. Another reason is that air compression with water depth affects the buoyancy of many marine animals, which complicates the DBA∼speed relationship depending on swim angle [94–96]. Furthermore, GPS is restricted to (potentially infrequent) resurfacing events for diving animals and so scaling DBA with GPS-derived speed is problematic for extended periods of time during underwater movements. Taken together, while we do not rule out extensions of the MVF method for use in such environments, we advocate that, in its current form, it is most suitable for evaluating movements on land.

**Figure 6.**
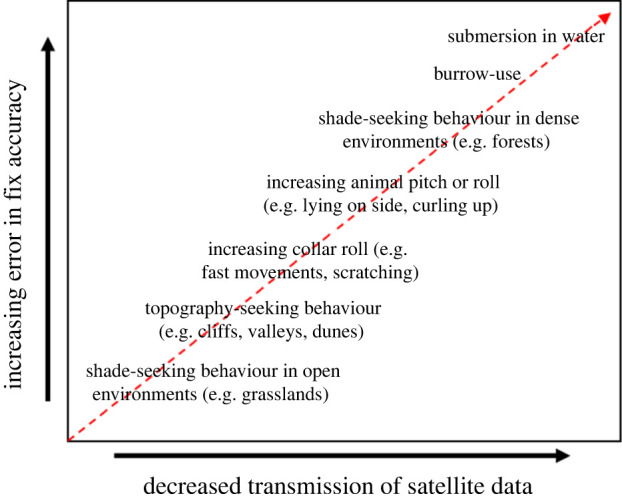
Schematic diagram illustrating the factors related to animal behaviour that can change the quality of GPS fixes.

Importantly, the validity of this method is dependent on the interaction between a focal species’ behaviour and where it inhabits—the critical limitation being the assumption that fixes are accurate during periods of moving. This is demonstrably not always the case (figure 6), even in our study area, the Kgalagadi Transfrontier Park, which is open, with relatively sparse vegetation. Since vegetation type and density are key modulators of GPS accuracy [36,39,40,44,50], the viability of our method needs to be tested within other (e.g. more vegetated) environments.

Nevertheless, for the study species in question, we have highlighted the effectiveness of this method and, in line with the above considerations, have demonstrated that a general correlation does exist between the magnitudes of both DBA and GPS speed during movement periods (figure 2; electronic supplementary material, figure S2.1:4). As such, we suggest that this approach could be used further to discern reliable events of high performance (e.g. hunt chases) and implemented within the dead-reckoning framework (see [67,97]), both as a corollary to the DBA–speed relationship (required for the speed coefficient) [67] and the GPS screening protocol prior to the correction process of dead-reckoned tracks [68]. At the very least, we demonstrate the utility of GPS speed to be included as a useful parameter for identifying behaviours and this may be of value to more complex approaches (e.g. machine learning (see [98,99–101]), the lowest common denominator (LoCoD) method [102] and space-state models (e.g. [103,104]) for precluding certain behaviours from movement and screening for location error. Indeed, applying this method as a validator of movement extent within behaviour-based studies over finely resolved space and time may facilitate the powers of inference, such as when considering animal responses to human barriers (see [105]). Lastly, we theorise that high fix frequency will help elucidate fix inaccuracy within areas of high canopy cover, possibly via extensions to this method such as including an upper GPS speed threshold limit and comparing variation in GPS speed juxtaposed to DBA estimates and GPS- and DD-derived heading estimates (see electronic supplementary material, S4).

## 5. Conclusion

Here, we reaffirm the importance of initial GPS screening to avoid inaccurate movement estimates. Animal behaviour seems to be a major modulator of GPS performance, and this is particularly germane in collared species due to the interaction between behaviour and collar orientation. The proposed MVF method provides a basis for high-resolution GPS screening, which is user friendly, computationally quick and focuses on identifying behaviour to filter GPS data. Movement-defined thresholds can be modelled according to the focal species in question, while further differences between motion sensor and GPS derivatives can be incorporated into this MVF foundation to resolve fix inaccuracy during movement. Movement-based outputs comparing MVF values from lion data exemplified the degree of inaccuracy associated with GPS jitter and the importance of removing such additive error prior to assessing fine-scale trends of movement, particularly step length. Our results show that consideration of data from both GPS units and motion sensors greatly helps validate true movement patterns and reaffirms the caution required when interpreting fine-scale GPS sampling such as during ARS analysis. Further work could assess the value of MVF for other species with different activities and habitat selections, particularly those that move within highly vegetated areas. The consequences of the errors introduced by GPS inaccuracies are broad, including erroneous inferences of behaviour, movement, speed and energy budgets. The approach proposed here avoids these errors and enables accurate assessments of these traits.

## Data accessibility

We provide a step-by step R script for implementing the MVF method on an example lion dataset (containing DD and GPS data files). The R script and example dataset is available on GitHub (available at [106]).

The data are provided in the electronic supplementary material [107].
